# Suture Button versus Screw Fixation for Distal Tibiofibular Injury and Expected Value Decision Analysis

**DOI:** 10.7759/cureus.19890

**Published:** 2021-11-25

**Authors:** Spencer S Schulte, Scott L Oplinger, Hunter R Graver, Kyle J Bockelman, Landon S Frost, Justin D Orr

**Affiliations:** 1 Orthopaedics, William Beaumont Army Medical Center, El Paso, USA; 2 F. Edward Hebert School of Medicine, Uniformed Services University of the Health Sciences, Bethesda, USA

**Keywords:** outcome, suture button, syndesmosis, fracture, ankle

## Abstract

Patient preference for fixation technique of syndesmotic injury in the presence of an ankle fracture is not known. This study followed a five-step process for expected value decision analysis: decision tree, outcome probabilities, expected patient values, foldback analysis, and sensitivity analysis. Outcome variables were “well” (cases that did not require further procedures or suffer any complications related to surgery), surgical site infection (SSI), loss of reduction (LOR), hardware removal (HWR), and malreduction. The systematic review included 22 studies including 358 patients who underwent suture button fixation and 739 who underwent screw fixation. Outcome probabilities for suture button fixation were 76.4% well, 6.2% SSI, 5.4% LOR, 10.4% HWR, and 1.6% malreduction. Outcome probabilities for screw fixation were 47.1% well, 4.3% SSI, 8.1% LOR, 30.7% HWR, and 9.8% malreduction. After the survey and foldback analysis, overall utility values for suture button and screw fixation were 7.46 and 4.78, respectively. One-way sensitivity analysis revealed that the overall utility value for suture button fixation was greater than the utility value of screw fixation under all circumstances except when the rate of malreduction for suture button fixation was theoretically elevated to 85%. Level of evidence: therapeutic, level IV.

## Introduction and background

Ankle fractures are one of the most common injuries treated by orthopedic surgeons and 20% of ankle fractures that require operative fixation have syndesmotic disruption [1,2]. Restoration and fixation of the syndesmosis are necessary to maintain a reduced tibiotalar joint and restore patient function [3]. Among many described techniques, screws and suture buttons are the preferred methods of syndesmotic fixation. Many studies of varying quality have evaluated the functional outcomes after screw fixation or suture button fixation of the syndesmosis and the differences between the two [4-27]. Despite the commonality of the injury and the volume of literature produced on the subject, no consensus on syndesmosis fixation has emerged.

In such a situation where treatment is dictated by surgeon preference, expected value decision analysis is a tool that can be used to incorporate patient preference in complex medical decision-making [28-30]. This method integrates published probabilities of outcomes in the literature with patient values with regard to what postoperative outcomes he or she prefers. This yields quantitative expected values for each outcome variable.

The purpose of this study is to determine whether patients prefer suture button or screw fixation for syndesmotic disruption. We hypothesize that patients have a greater expected value for suture button fixation due to the lower rate of implant removal and a lower rate of postoperative loss of reduction with suture button fixation.

## Review

Search strategy

This study was conducted in accordance with the Preferred Reporting Items for Systematic Reviews and Meta-Analyses (PRISMA) criteria. An advanced search was conducted in the PubMed database using the phrases “syndesmosis fixation,” and “screw versus suture button fixation.” Results were filtered to include only abstracts, case reports, clinical trials, journal articles, meta-analyses, multicenter studies, randomized controlled trials, reviews, and systematic reviews published in English prior to February 01, 2020. The last search was conducted on February 01, 2020.

Selection criteria

Level I-IV studies were included that reported a minimum of one outcome measure for fixation of unstable ankle fractures with accompanying distal tibiofibular syndesmosis injuries treated with either syndesmotic screw or suture button fixation. Outcomes were then extracted from each study and pooled to determine an outcome percentage. Outcomes that were reported in more than two studies were included in the questionnaire. Conference abstracts, purely biomechanical studies, articles that did not report postoperative outcomes, and those that examined non-generalizable subpopulations (e.g., diabetic patients, those who had missed syndesmotic injuries, etc.) were excluded.

Data extraction and quality assessment

Two independent reviewers screened each article’s title and abstract. Predefined outcomes of interest included surgical site infection (SSI), loss of reduction (LOR), need for hardware removal (HWR), and malreduction of the syndesmosis. The quality of nonrandomized studies was assessed utilizing the Newcastle-Ottawa Quality Assessment Scale, and the quality of randomized studies was assessed with the Cochrane tool for bias risk assessment (Tables 1, 2, respectively) [31,32].

**Table 1:** The Newcastle-Ottawa Quality Assessment (NOQA) Scale for cohort studies. Studies with a score of 9-10 points are considered very good, 7-8 points good, 5-6 points satisfactory, and 0-4 points unsatisfactory.

	Selection of study group	Comparability of groups	Outcome	Total score
Cottom et al. 2009 [6]	4	2	3	9
Egol et al. 2010 [8]	4	2	3	9
Hamid et al. 2009 [9]	4	2	3	9
Kocadal et al. 2016 [11]	4	2	3	9
Moore et al. 2006 [15]	4	2	3	9
Naqvi et al. 2012 [16]	4	2	3	9
Rigby and Cottom 2013 [19]	3	2	3	8
Seyhan et al. 2015 [20]	4	2	3	9
Thornes et al. 2005 [22]	4	2	3	9
Tucker et al. 2013 [23]	4	2	3	9
Walker et al. 2015 [24]	3	1	3	7
Weening et al. 2005 [25]	3	2	3	8
Degroot et al. 2011 [7]	N/A			Case series
Manjoo et al. 2010 [14]	N/A			Case series
Qamar et al. 2011 [18]	N/A			Case series
Storey et al. 2012 [21]	N/A			Case series
Willmott et al. 2009 [26]	N/A			Case series

**Table 2:** Cochrane risk bias assessment tool for randomized controlled trials. “+” indicates that the domain was satisfied. “-” indicates that the domain was not satisfied. If a study had a risk of bias in multiple domains (as indicated by either “-” or “unclear”), then the study might be judged to be at high risk of bias overall.

	Random sequence generation	Allocation concealment	Blinding of participants and personnel	Blinding of outcome assessment	Incomplete outcome data	Selective reporting	Other bias
Andersen et al. 2018 [4]	+	+	Unclear	+	+	+	+
Coetzee and Ebeling 2008 [5]	Unclear	Unclear	-	Unclear	+	+	+
Kortekangas et al. 2015 [12]	+	+	-	Unclear	+	+	+
Laflamme et al. 2015 [13]	+	+	-	+	+	+	+
Høiness et al. 2004 [10]	+	-	-	-	+	+	+

Expected value decision analysis

This study followed the five-step process for expected value decision analysis: the creation of a decision tree, determining outcome probabilities, determining expected patient values, foldback analysis, and sensitivity analysis. We sought to determine patient preference between the use of screws or suture button constructs for fixation of injuries to the ankle syndesmosis. Five separate outcome variables were determined for each treatment choice, including a “well” category, SSI, LOR, HWR, and malreduction of the syndesmosis. The “well” category was determined to be cases that did not require further procedures or suffer any complications related to the surgery. For example, if a syndesmotic screw breakage did not result in any other definable complication or a return to the operating room, this patient would be counted in the “well” category. SSI included both deep and superficial infections requiring either operative or nonoperative treatment. LOR was determined as diastasis or displacement of the syndesmosis after initially being determined as reduced at the time of the surgery. HWR included any cases that had either planned or unplanned removal of the syndesmosis fixation hardware. Malreduction was defined by each included study but generally determined to be greater than 2 mm of difference in fibular station between operative and nonoperative limbs. Malreduction was determined postoperatively by independent reviewers during the primary study. This was determined by plain radiographs with or without contralateral ankle radiographs, and in some studies by computed tomography (CT).

Foldback analysis was then performed to determine the overall utility value of each treatment. This was achieved by multiplying each outcome probability by the mean patient response. This yielded a utility value for each outcome variable. The sum of these values was the overall utility value. The treatment with the higher utility value was considered to be superior.

Sensitivity analysis was then performed by varying the outcome probabilities for each outcome variable (SSI, LOR, HWR, and malreduction) from 0% to 100% while keeping all other variables constant. Given that the suture button had a higher utility value, we varied these outcome probabilities in the suture button variables only to determine at what point the suture button utility value was equal to or lower than the screw utility value.

Results

**Figure 1:**
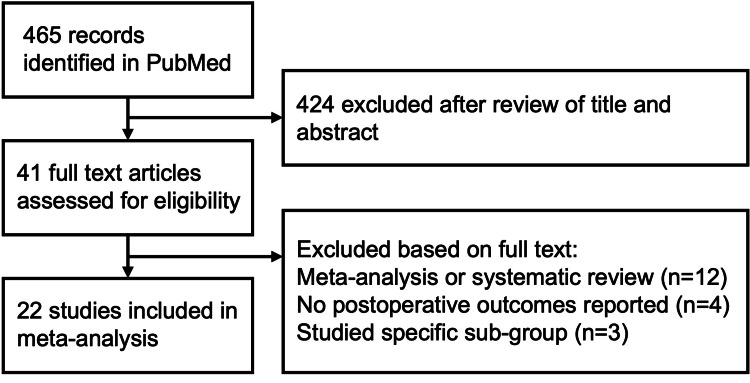
Study selection flowchart.

The literature search returned 465 results (Figure 1). After review of the title and abstract, 424 results were excluded and 41 full-text articles were assessed for eligibility. Of these 41 articles, 12 meta-analyses and systematic reviews were excluded, four articles were excluded due to no postoperative outcomes reported, and three articles were excluded due to the inclusion of nongeneralizable subpopulations. This resulted in 22 papers meeting our inclusion criteria, including five randomized controlled trials and eleven comparative studies, with six studies investigating screw fixation only and five studies investigating suture button fixation only [5-17,20-28]. A total of 1,097 subjects were included in these studies, with outcomes reported for 358 subjects who underwent suture button fixation 739 patients who underwent screw fixation.

Expected patient outcome variables were then determined by surveying 100 consecutive volunteers. Volunteers included all adult patients seen in an outpatient orthopedic clinic setting. To limit bias, patients with prior syndesmosis injury and treatment were excluded. Each volunteer was provided with a questionnaire with 10 different questions detailing how likely they were to prefer a treatment based on the percentage of each of the five outcome variables. These questions asked patients to scale their preference from 0 to 10, with 10 being the most likely to prefer that treatment. Participation was voluntary and anonymous. After 100 questionnaires were collected, the average response for each question was determined. The questionnaire is shown in Table 3.

**Table 3:** Questionnaire.

Please provide a number to answer the following questions. DO NOT identify yourself on this sheet. When finished, place your answer sheet in the box. Thank you for your time in helping with this study. Your contribution will help expand the knowledge base of Orthopedic Surgery and improve patient outcomes.
Syndesmosis Injury
Background: Syndesmotic injuries occur when the ligamentous structure between the tibia (shin bone) and the fibula (the little bone on the outside of the shin bone) becomes disrupted above the ankle. There are currently two ways of surgically treating this: using a metal screw or strong thread with buttons on each end. We are trying to find out which surgical option is better.
To help you understand the survey, the term “well” means essentially the perfect surgery with no infection, no need for another surgery, and no problem with putting the bones back in the right position. The term “surgical site infection” means there is an infection that requires antibiotics and/or repeat surgery to clear the infection. “Malreduced” means that during the surgery the bones were not put back together properly. “Loss of reduction” means that the bones were put together properly at the time of surgery, but that some time after surgery the hardware lost its ability to hold the bones together properly. To remove an implant requires an additional surgery.
1: On a scale of 0–10 (10 being the most you would want to have the specific treatment listed), how much would you like to have a surgery that has a 76.4% chance of being “well”?
2: On a scale of 0–10 (10 being the most you would want to have the specific treatment listed), how much would you like to have a surgery that has a 47.1% chance of being “well”?
3: On a scale of 0–10 (10 being the most you would want to have the specific treatment listed), how much would you like to have a surgery that has a 6.2% chance of having a “surgical site infection”?
4: On a scale of 0–10 (10 being the most you would want to have the specific treatment listed), how much would you like to have a surgery that has a 4.3% chance of having a “surgical site infection”?
5: On a scale of 0–10 (10 being the most you would want to have the specific treatment listed), how much would you like to have a surgery that has a 5.4% chance of “loss of reduction”?
6: On a scale of 0–10 (10 being the most you would want to have the specific treatment listed), how much would you like to have a surgery that has a 8.1% chance of “loss of reduction”?
7: On a scale of 0–10 (10 being the most you would want to have the specific treatment listed), how much would you like to have a surgery that has an 10.4% chance of needing the implant removed?
8: On a scale of 0–10 (10 being the most you would want to have the specific treatment listed), how much would you like to have a surgery that has a 30.7% chance of needing the implant removed?
9: On a scale of 0–10 (10 being the most you would want to have the specific treatment listed), how much would you like to have a surgery that has a 1.6% chance of being “malreduced”?
10: On a scale of 0–10 (10 being the most you would want to have the specific treatment listed), how much would you like to have a surgery that has a 9.8% chance of being “malreduced”?

We performed a systematic review of these sources and extracted the probability for all outcomes reported. Seventeen studies reported outcome scores (thirteen American Orthopaedic Foot & Ankle Society [AOFAS], four Olerud-Molander index, and two reported both). A total of 11 studies reported data on SSIs. Eight studies reported on malreduction. Seven studies reported on the postoperative LOR. Six studies reported time to weight-bearing. Other outcome variables that were reported by two or fewer studies included synostosis, radiographic osteoarthritis, return to sport, and return to work. Outcome probabilities, mean patient responses, and utility values are reported in Tables 4, 5.

**Table 4:** Outcome probabilities and mean survey response for suture button fixation. SB: suture button; SSI: surgical site infection; LOR: loss of reduction; HWR: hardware removal

Outcome	SB probability (%)	SB mean response	SB utility value
Well	76.4	7.96	6.08
SSI	6.2	5.46	0.34
LOR	5.4	6.62	0.36
HWR	10.4	5.69	0.59
Malreduction	1.6	5.4	0.09

**Table 5:** Outcome probabilities and mean survey response for screw fixation. SSI: surgical site infection; LOR: loss of reduction; HWR: hardware removal

Outcome	Screw probability (%)	Screw mean response	Screw utility value
Well	47.1	4.89	2.30
SSI	4.3	6.29	0.27
LOR	8.1	4.52	0.37
HWR	30.7	4.85	1.49
Malreduction	9.8	3.58	0.35

**Figure 2:**
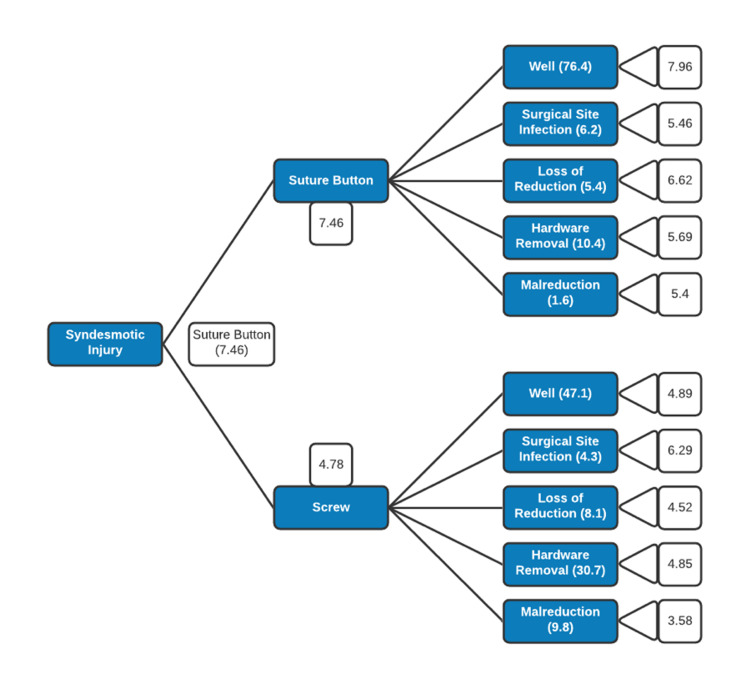
Decision tree. From left to right, the shown values are the overall utility value, outcome probability, and mean patient response. With a utility value of 7.91, the suture button is determined to be the superior option.

Foldback analysis yielded an overall utility value for the suture button construct of 7.46. The overall utility value of the screw construct was 4.78. Based on these utility values, the suture button construct was determined to be the favored option. The decision tree summarizing these findings is shown in Figure 2.

**Figure 3:**
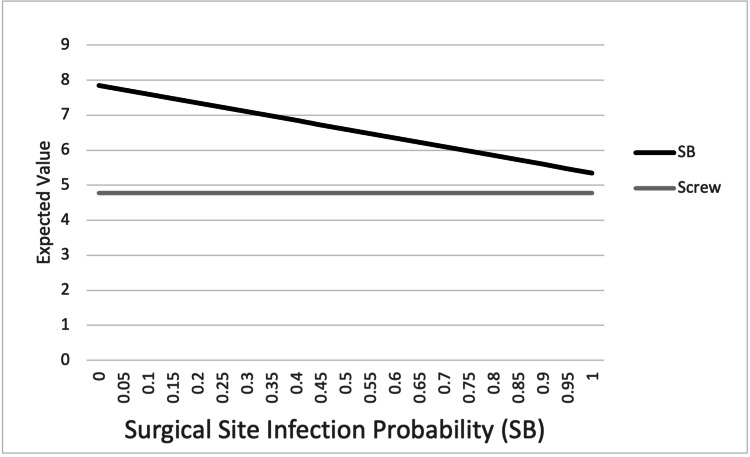
Graphical representation of one-way sensitivity analysis for surgical site infection. Top line: SB; bottom line: screw. SB: suture button

**Figure 4:**
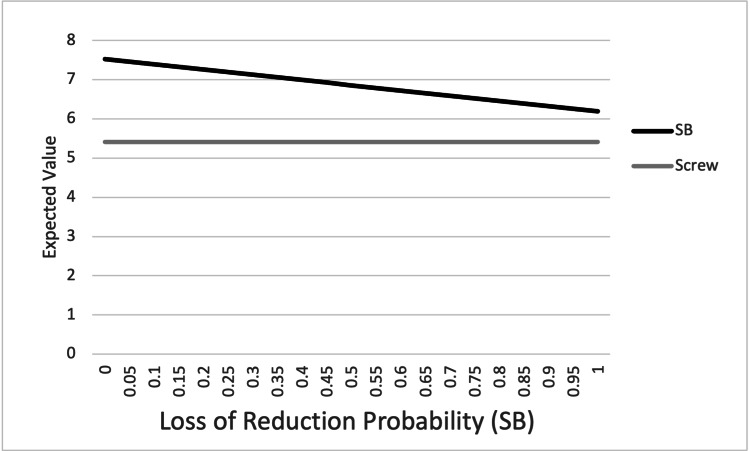
Graphical representation of one-way sensitivity analysis for loss of reduction. Top line: SB; bottom line: screw. SB: suture button

**Figure 5:**
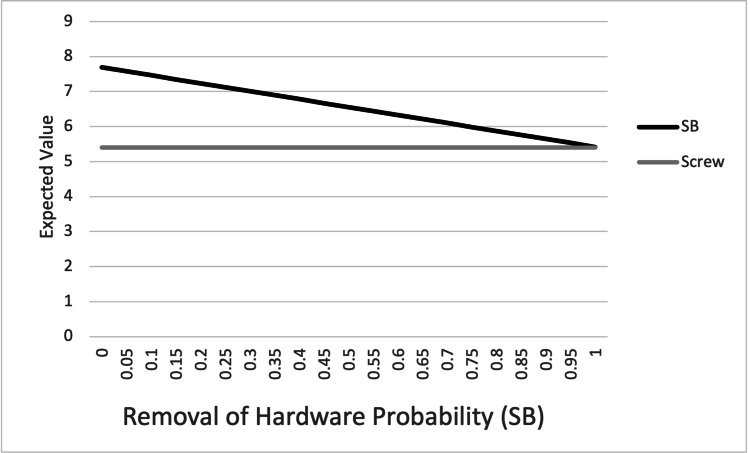
Graphical representation of one-way sensitivity analysis for removal of hardware. Top line: SB; bottom line: screw. SB: suture button

**Figure 6:**
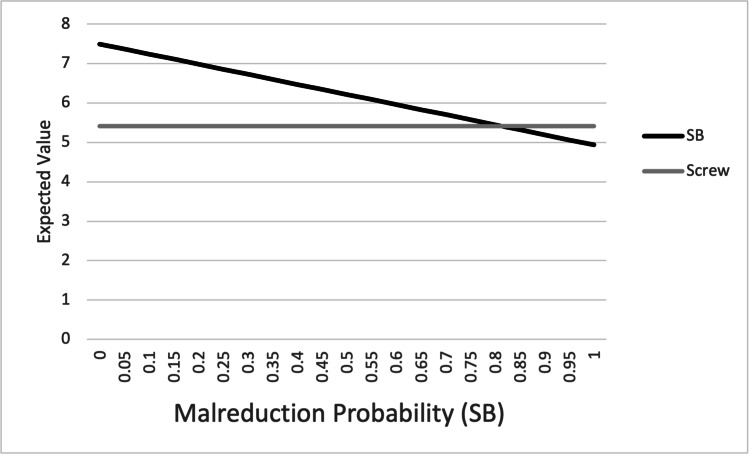
Graphical representation of one-way sensitivity analysis for malreduction. Top line: SB; bottom line: screw. SB: suture button

One-way sensitivity analysis was performed by varying the probability of the four outcome variables (SSI, LOR, HWR, and malreduction) in the suture button construct from 0% to 100% (Figures 3-6). For SSI, LOR, and HWR, the overall utility value of the suture button construct never decreased to or below the utility value of the screw construct (4.78). When considering the rate of malreduction (1.6% for suture button vs 9.8% for screw), suture button fixation is favored until the rate of malreduction for suture button fixation is varied to 85% and above; only at this theoretical high rate of malreduction is the utility value of screw fixation equal to or superior to suture button fixation.

Discussion

In the presence of an operatively treated ankle fracture, syndesmotic fixation strategy is a debated topic with no clear consensus. In multiple recent systematic reviews, the suture button has been favored in terms of cost-effectiveness, outcome scores, and maintaining reduction of the syndesmosis [17,27]. Our literature review and patient preferences were in accordance with the prior literature. It is important to note that six of the 22 studies included in our review reported a financial conflict of interest [4,12,14,19,22,25]. The rate of SSI our review found (6.2% suture button vs. 4.3% screw) was similar to that found by Onggo et al. (4.3% suture button vs 2.9% screw); however, our study pooled the data from 205 subjects while Onggo et al. included 140 subjects. We included level III and IV studies whereas Onggo et al. excluded these studies. We decided to include lower-level studies to maximize the number of subjects included for each outcome variable as well as our ability to report on multiple variables. The rate of malreduction we found (1.6% suture button vs. 9.8% screw) is similar to that reported by Zhang et al. (1.0% suture button vs. 12.6% screw). The rate of hardware removal our study found (10.4% suture button vs. 30.7% screw) was similar to that found in prior reviews [27].

The authors of the primary studies included in this review used variable definitions of the reported outcomes. For example, hardware removal may be planned or anticipated and not included as an adverse outcome. The rates of hardware removal in screw fixation were consistently higher than those of suture button fixation. We included any hardware removal in our pooled outcome for the questionnaire and found that suture button fixation maintained a superior utility value even when the rate of suture button hardware removal was theoretically elevated above that of screw fixation removal.

In our literature review, there were variables reported that favored suture button fixation that were not amenable to the methodology of our questionnaire: suture button was favored in terms of the AOFAS score (suture button 92.0 vs. screw 86.3) and time to weight-bearing (suture button 38.3 days vs. screw 66.0 days). This was in accordance with prior systematic reviews that found AOFAS scores to favor suture button fixation [17,27]. Outcome scores were excluded from our question because they are not reported as percentages and were not amenable to our methodology of foldback and one-way sensitivity analyses. If it were possible to include outcome scores in our analysis it would cause suture button fixation to be favored to a greater magnitude.

Foldback analysis validated our hypothesis that suture button fixation was viewed by patients as being superior to screw fixation with an overall value of 7.46 versus 4.78, respectively. The greatest difference in utility value was attributed to the “well” category (6.08 suture button vs. 2.30 screw). This suggests that patients place the greatest value in their surgery going well and not having to return to the operating room for a second surgery. The second greatest difference in utility value was found for hardware removal (0.59 suture button vs. 1.49 screw). There was also a large difference in utility value for malreduction (suture button 0.09 vs. screw 0.35). Both of these variables indicate patients placed a large value in not having to return to the operating room. The magnitude of difference in probability for these three variables (“well”, HWR, and malreduction) was the greatest among all variables. We hypothesize that the difference in probabilities paired with the need to return to the operating room led to the difference in perceived value for these variables.

Limitations of our study are rooted in our literature review and the nature of the questionnaire. Though studies examining the complication profile of syndesmotic fixation are robust, these studies lack uniformity which makes it difficult to make recommendations based on the body of literature. These studies lack uniformity in how syndesmotic injury is determined, how it is treated (how the syndesmosis is reduced, number of screws used, number of cortices crossed with screws, number of suture buttons used), accompanying ankle fracture (isolated syndesmotic injury in the absence of malleolar fractures, isolated lateral malleolus fracture, bimalleolar ankle fracture, trimalleolar ankle fracture), how reduction and malreduction is determined (CT versus plain films), and heterogeneity of reported outcomes. Another area of limitation for our study was that patients may not fully understand the nature of a syndesmotic injury and the treatment thereof. Subjects were allowed only the information present on the questionnaire. The purpose of limiting further discussion was to limit the possibility that an individual orthopedic surgeon could impart his or her own treatment bias to the subjects of the questionnaire. The value of a decision analysis study is that it abstracts a general patient’s preference given the body of literature aside from the bias that an individual surgeon may introduce. Patients’ prior surgical history and demographics were variables in the study which could introduce bias based on prior interventions or health states. To limit this potential area of bias, we excluded subjects from this study who had had prior foot and ankle surgeries or conditions.

## Conclusions

Decision analysis supported our hypothesis that patients have a greater expected value for suture button fixation given the lower rate of implant removal, the lower rate of postoperative LOR, and the lower rate of intraoperative malreduction. Our findings paired with the findings of several systematic reviews suggest that clinicians should give greater consideration for suture button fixation of the syndesmosis. Our study provides additional contextual information for surgeons when counseling patients in shared decision-making for their orthopedic injuries.
